# Role of Non-coding Regulatory RNA in the Virulence of Human Pathogenic *Vibrios*

**DOI:** 10.3389/fmicb.2016.02160

**Published:** 2017-01-11

**Authors:** Diliana Pérez-Reytor, Nicolás Plaza, Romilio T. Espejo, Paola Navarrete, Roberto Bastías, Katherine Garcia

**Affiliations:** ^1^Centro de Investigación Biomédica, Facultad de Ciencias de la Salud, Instituto de Ciencias Biomédicas, Universidad Autónoma de ChileSan Miguel, Chile; ^2^Institute of Nutrition and Food Technology, University of ChileSantiago, Chile; ^3^Laboratory of Microbiology, Institute of Biology, Pontificia Universidad Católica de ValparaísoValparaíso, Chile

**Keywords:** sRNA, virulence, quorum sensing, pathogenic vibrios, *Vibrio cholerae*, *Vibrio vulnificus*, *Vibrio parahaemolyticus*, bioinformatics analysis

## Abstract

In recent decades, the identification of small non-coding RNAs in bacteria has revealed an important regulatory mechanism of gene expression involved in the response to environmental signals and to the control of virulence. In the family *Vibrionaceae*, which includes several human and animal pathogens, small non-coding RNAs (sRNAs) are closely related to important processes including metabolism, quorum sensing, virulence, and fitness. Studies conducted *in silico* and experiments using microarrays and high-throughput RNA sequencing have led to the discovery of an unexpected number of sRNAs in *Vibrios*. The present review discusses the most relevant reports regarding the mechanisms of action of sRNAs and their implications in the virulence of the main human pathogens in the family *Vibrionaceae: Vibrio parahaemolyticus, V. vulnificus* and *V. cholerae*.

## Introduction

The family *Vibrionaceae* is composed of a set of Gram-negative γ-proteobacteria present in marine environments (Heidelberg et al., 2002). The members of this family are easily isolated from different niches such as estuaries or deep water. They can also be found in planktonic form or on biotic and abiotic surfaces, establishing symbiotic or pathogenic interactions (Sun et al., 2013). The family includes important pathogens in aquaculture, such as *Vibrio anguillarum*, *V. salmonicida*, and *V. harveyi* (Thompson et al., 2004; Zhang et al., 2012). However, the most clinically significant members of the genus *Vibrio* are the human pathogens *V. cholerae*, *V. vulnificus* and *V. parahaemolyticus* (Thompson et al., 2004).

Most studies concerning the family *Vibrionaceae* have focused on *V. cholerae*, the species responsible for several outbreaks of cholera in places such as Bangladesh, Kolkata, and more recently Haiti (Antonova and Hammer, 2015). Today it is known that this microorganism produces a series of virulence factors that facilitate its colonization in the intestine, allowing a subsequent illness characterized by watery diarrhea, often fatal if untreated (Bradley et al., 2011; Cheng et al., 2015). The main virulence factors are the cholera toxin (CT) and the toxin co-regulated pilus (TCP; Bradley et al., 2011; Sengupta et al., 2016).

A second pathogen, *V. vulnificus*, is closely related to *V. cholerae* and is considered an opportunist pathogen that can cause septicemia in humans, frequently with fatal results (Alice et al., 2008). Infections produced by this pathogen represent 95% of all deaths related to the consumption of seafood in the United States, with a mortality rate close to 50%. As a result, it is considered the most lethal food-transmitted pathogen in that country, and possibly in the world (Oliver, 2015). The main factor for its virulence is the presence of an antiphagocytic capsule (Thompson et al., 2004; Oliver, 2015). Finally, a third bacterial pathogen associated with the consumption of partially cooked or raw seafood is *V. parahaemolyticus* (Raghunath, 2015). This species is composed of numerous strains widely distributed in marine environments throughout the world (García et al., 2009, 2013; Loyola et al., 2015) but only a few cause diarrhea in humans. Virulent *V. parahaemolyticus* strains have different virulence factors, including adhesins, thermostable direct hemolysin (TDH) and TDH related hemolysin (TRH), as well as two different type-III (T3SS1 and T3SS2) and type-VI (T6SS1 and T6SS2) secretion systems, one in each of its two chromosomes (Letchumanan et al., 2014).

The infection process of these pathogenic bacteria requires the regulation of an arsenal of virulence factors that allow the bacteria to invade and survive inside their hosts, where they must acquire nutrients and resist environmental tension generated by the defenses of the host (Porcheron and Dozois, 2015). Recently, a new form of genetic regulation in bacteria has been revealed with the identification of small non-coding RNAs (sRNAs; Bardill and Hammer, 2012; Michaux et al., 2014). These RNAs form a heterogeneous group with 50 to 500 nucleotides (nt) that act via different mechanisms to modulate a wide range of cell responses (Repoila and Darfeuille, 2009; Silveira et al., 2010; Song et al., 2014), including regulation of virulence genes (Papenfort and Vogel, 2014). The regulation performed by these molecules can occur at all levels of gene expression, from the initiation of transcription, through translation control, to protein activity (Waters and Storz, 2009; Papenfort and Vogel, 2014). This control is achieved through a variety of mechanisms, including changes in RNA conformation, protein binding and RNA-RNA or RNA-DNA base-pairing (Waters and Storz, 2009; Oliva et al., 2015). Recent studies by massive RNA sequencing (RNA-seq) have revealed the wide distribution and large number of sRNAs present in bacterial genomes. But there is still a long way to go to understand the function of these molecules in the different networks including virulence regulation (Papenfort and Vogel, 2010; Sorek and Cossart, 2010).

The global prevalence of pathogens against humans and animals within *Vibrio* strengthens the need to understand how sRNAs participate in the regulation of different virulence factors associated with these bacteria and their effects on hosts (Letchumanan et al., 2014). This review discusses the most relevant reports regarding the mechanisms of action of sRNAs and their implications in the virulence of the main human pathogens in the family *Vibrionaceae: V. parahaemolyticus*, *V. vulnificus* and *V. cholerae*.

## Classification Of sRnas

Most sRNAs described to date can suppress certain cellular processes, inhibiting translation and decreasing the expression of the target gene, or acting on both processes simultaneously. However, it has also been documented that sRNAs can activate gene expression (Papenfort and Vanderpool, 2015). The main regulation mechanisms used by sRNAs described to date can be classified into five categories, as follows:

### sRNAs that Modulate Protein Activity

Small non-coding RNAs that modulate protein activity are *non-base-pairing* molecules and can interact directly with proteins to influence their activity by sequestration (Michaux et al., 2014). The system understood in this context is the global carbon storage regulator in the CsrA/RsmA family (Carbon storage regulator/Regulator of secondary metabolism; Lenz et al., 2005).

CsrA/RsmA proteins are global regulators of gene expression at a post-transcription level and play an important role in the expression of virulence factors in several proteobacterial pathogens (Babitzke and Romeo, 2007; Vakulskas et al., 2015). CsrA binds to mRNA, commonly inhibiting translation by blocking the ribosome binding site (RBS). This leads to the rapid degradation of their target mRNA and is fundamental to the regulation of some specific virulence systems required for host infection (Vakulskas et al., 2015). The activity of CsrA is regulated by sRNAs of the CsrB family (100 to more than 400 in length). These sRNA remove this protein from the target mRNAs by binding to different sites (Romeo et al., 2013). These sRNAs indirectly activate genes suppressed by CsrA, antagonizing the activity of this protein, and can be found in multiple copies per genome, performing redundant functions (Papenfort and Vanderpool, 2015).

### sRNAs that Are Transcribed Away from Their Targets (Coded in *trans*)

Small non-coding RNAs that act in *trans* (sRNA-*trans*) are between 50 and 300 nt in length; they are imperfectly complementary to one or more regions of the target mRNA, which is coded in another region of the genome. sRNA-*trans* participate in the response of the cell to changes in environmental conditions and are also involved in the regulation of virulence in pathogenic species (Malecka et al., 2015). The general mechanism of sRNA-trans is to occupy the RBS of a target mRNA by base pairing with the Shine-Dalgarno (SD) sequence or the start codon, although they can also interact with the mRNA coding sequence (Feng et al., 2015). As they are only partially complementary with their target, sRNA-trans generally require a chaperone, Hfq, to facilitate RNA-RNA interactions due to limited complementarity between the sRNA-target mRNA and to strengthen its regulation (Wagner, 2013; Van Puyvelde et al., 2015). Hfq belongs to the group of Sm-like (LSm) proteins which are also found in eukaryotes and archaea (Vogel and Luisi, 2011; Papenfort and Vanderpool, 2015). It may actively remodel the RNAs to melt inhibitory secondary structures and also may serve passively as a platform to allow sRNAs and mRNAs to sample potential complementarity (Waters and Storz, 2009). Besides, Hfq is frequently required to protect the sRNAs from cellular ribonucleases such as RNase E (Papenfort and Vanderpool, 2015), and for many intracellular and extracellular pathogens it is known that Hfq deficiency dramatically affects virulence and fitness (*Escherichia coli*, Kulesus et al., 2008; *V. alginolyticus*, Deng et al., 2016; *Klebsiella pneumoniae*, Chiang et al., 2011; *Pseudomonas aeruginosa*, Sonnleitner et al., 2003; *Bordetella Pertussis*, Bibova et al., 2013).

In case of sRNA-*trans*, a single mRNA can be regulated by more than one sRNA (Bardill and Hammer, 2012; Duval et al., 2014; Chang et al., 2015). This type of sRNA has been more widely studied and is the most abundant type of sRNA discovered to date. An example of this group are the sRNAs called Qrr (*Quorum Sensing Regulatory* RNA) that are involved in the regulation of bacterial quorum sensing (QS; Silveira et al., 2010). These Qrr sRNAs regulate target mRNAs either positively or negatively affecting translation, stability and/or processing of the target mRNA (Bardill and Hammer, 2012; Feng et al., 2015; Van Puyvelde et al., 2015). In the case of negative regulation, the sRNA-*trans* is paired close to the RBS of the target mRNA, blocking translation. In many cases this generates recruitment of RNase E and subsequent degradation of the mRNA. In positive regulation, the coded sRNA-*trans* performs an anti-antisense base-pairing in the 5′ mRNA region. The ribosome binding site is thus revealed, promoting the stabilization of the target mRNA and hence translation. In this case, Hfq binds with both sRNA-*trans* and its corresponding mRNA target mediates the interaction (Fröhlich and Vogel, 2009; Feng et al., 2015). This can decrease the constant of apparent dissociation between the sRNA and its target mRNA and stabilize the sRNAs, protecting them from degradation by the RNase E (Bardill and Hammer, 2012; Duval et al., 2014).

### 5′ UTR Elements

The regulator elements of 5′UTR (untranslated regions) are sequences that act in *cis* in the mRNA and have gained special attention in recent years. This group of elements is associated with the detection of a wide range of signals, including temperature (so-called thermoswitches; Nguyen and Jacq, 2014) and riboswitches (Smith and Strobel, 2011). The latter control gene expression by direct binding to ions or small molecules such as metabolites, co-enzymes, amino acids and nucleotide bases, causing changes in the secondary structure of the transcript (Cressina et al., 2011; Furukawa et al., 2012).

### sRNAs Coded in the Opposite Chain of the Regulated RNA (Coded in *cis*)

Small non-coding RNA-*cis* are complementary to their target mRNA, and thus can interact autonomously. Their size also varies from approximately 100 to more than 300 nt, and may overlap with the 5′ or 3′ ends or be in a middle zone of the gene coded opposite the sRNA (Chang et al., 2015). These sRNAs are located in the antisense strand of their target RNA regions and their mechanism of action leads to the post-transcriptional down-regulation of the target gene through perfect extended duplex (Oliva et al., 2015).

In terms of their mechanisms of action, it has been proposed that sRNAs-*cis* may have several advantages over their homologs that act in *trans*. Full and lengthy complementarity between the sRNA and its target, observed in sRNA-*cis*, can lead to very stable duplexes, allowing regulation that is independent of proteins such as Hfq (Chang et al., 2015). Also, the *cis* position has the effect that sRNA and target are transcribed in close proximity, which can facilitate and improve interactions between the regulator molecules and their objective (Georg and Hess, 2011; Chang et al., 2015).

### The CRISPR/Cas System (Clustered Regularly Interspaced Short Palindromic Repeats)/CRISPR Associated

CRISPR/Cas systems provide an adaptive immune mechanism in bacteria and archaea (Barrangou et al., 2007; Westra et al., 2014) which recognizes and degrades exogenous nucleic acid belonging to viruses and plasmids that invade the cell (Rath et al., 2015). The cleavage of foreign nucleic acid occurs through Cas proteins specifically guided by sRNA of approximately 30 nt (Makarova et al., 2011). These guide sRNAs, called CRISPR RNAs (crRNAs), are found in a specific locus where each DNA sequence is flanked by a palindromic repeated sequence that participates in its genetic expression (Makarova et al., 2011; Rath et al., 2015). The immunological memory against future encounters with foreign nucleic acid is due to the incorporation of fragments of exogenous genetic material into the genome of the bacterium (Plagens et al., 2015; Rath et al., 2015). These systems are very diverse in terms of number, genetic structure and mechanism of action of Cas proteins (Makarova et al., 2011). Makarova et al. (2011) proposed a new classification of CRISPR/Cas systems of five types (types I–V) divided into two major classes. Class 1 systems have multi-subunit crRNA–effector complexes, whereas Class 2 system functions are carried out by a single subunit crRNA–effector module (Makarova et al., 2011).

## sRnas Involved In The Virulence Of Human Pathogenic *Vibrio* spp.

It has been documented that sRNAs regulate several important processes in bacterial pathogens (Michaux et al., 2014; Sabharwal et al., 2015), including homeostasis of the outer membrane, QS, adaptation to stress, iron homeostasis, biofilm formation, virulence and host-cell contact, as well as development and metabolism (Liu et al., 2009; Papenfort and Vogel, 2014; Papenfort and Vanderpool, 2015; Sengupta et al., 2016). However, in *Vibrios* only few sRNAs have been studied experimentally and even fewer have been related to virulence.

Several sRNAs have been found inactive in cells lacking Hfq protein, which has been described as a master regulator of gene expression in bacteria, essentially due to its ability to mediate the interaction of sRNAs with their mRNA targets, including those related to virulence in Gram-negative bacteria (Kim et al., 2015; Feliciano et al., 2016). The association between Hfq and virulence in *E. coli* was revealed by Kulesus et al. (2008). Strains carrying a disruption in *hfq* showed restricted motility, chemotaxis, ability to form biofilm and incapability to colonize effectively. The negative impact on all these processes reflects the capacity of this chaperone to interact with a wide range of different regulatory sRNA (Kulesus et al., 2008). Similarly, it has been reported that the deletion of the *hfq* gene in *Salmonella enterica* serovar Typhimurium produces a non-motile strain which is highly attenuated in its ability to infect mice, invade epithelial cells, secrete virulence factors and to survive and proliferate within macrophages. These significant phenotypes suggest that Hfq, as in *E. coli*, interacts with many sRNAs which are involved in virulence (Hébrard et al., 2012).

Hfq also appears to contribute in several distinct ways to the virulence of *V. cholerae*. It has been shown that Δ*hfq* strains fail to colonize the suckling mouse intestine (Ding et al., 2004), as well as deficient response to stress, changes in population behavior, altered metabolic regulation and loss of virulence (Shao and Bassler, 2012; Wagner, 2013). As Hfq often acts in conjunction with sRNAs, these molecules also play major roles in the control of cholera virulence (Ding et al., 2004).

One of these sRNA is VrrA (Vibrio regulatory RNA of OmpA; Sabharwal et al., 2015), whose expression is controlled by RpoE; it is capable of regulating the outer membrane porins OmpA and OmpT (Song et al., 2010; Sabharwal et al., 2015), the formation of outer membrane vesicles (OMV; Richard et al., 2010) and the expression of RbmC, which is important for biofilm formation (Sabharwal et al., 2015; Teschler et al., 2015). VrrA positively controls the release of OMV, causing an increase by repressing *ompA* (Song and Wai, 2009; Sabharwal et al., 2015). The formation and release of OMVs have gradually been more implicated in novel pathways of virulence factor delivery, since they have been proposed to play a role in several virulence mechanisms: periplasmic enzyme and transport, evasion of the immune system and toxin delivery. Additionally, attenuation in *V. cholerae* virulence was demonstrated using a deletion mutant of VrrA in an infant mouse model. Song et al. (2014) hypothesized that in the later stages of *V. cholerae* infection in the host, bacteria can move away from the epithelial surface into the intestinal lumen. During this time the bacteria may undergo a switch from attachment to the epithelial surface to detachment. This process may be associated with upregulation of VrrA, which can modulate expression of both a colonization factor (Tcp) and the attachment factor (RbmC; Song et al., 2014). VrrA mediates the downregulation of TcpA, a major virulence factor essential for host colonization in *V. cholerae*, possibly by binding directly to the 5′ UTR of *tcpA* (Song and Wai, 2009). The regulation of *rbmC* occurs by direct pairing with the 5′ end of the mRNA, inhibiting its translation and thus the formation of biofilms (Teschler et al., 2015). Notably, this regulation is not stringently dependent on Hfq protein, and hence VrrA represents the first example of direct regulation of sRNA on a biofilm matrix component, bypassing global master regulators (Song et al., 2014).

There are another two sRNAs, called TarA (ToxT activated RNA A; Richard et al., 2010) and TarB, which as their names imply are under the control of ToxT and involved in the regulation of virulence genes in *V. cholerae* (Bradley et al., 2011). Richard et al. (2010) observed that TarA, a highly conserved small regulatory RNA, unstable in the absence of Hfq and whose transcription is activated by ToxT, negatively regulates *ptsG*, a gene that codes the main glucose transporter in *V. cholerae*. The researchers showed that this sRNA influences glucose uptake by affecting the expression of PtsG though complementary bases between TarA and the 5′ region of the mRNA *ptsG*. However, the expression of TarA is activated by the virulence gene pathway in *V. cholerae* and not by glycolytic intermediates (Richard et al., 2010). These authors also showed that a classical *V. cholerae* O395 mutant lacking TarA is compromised for infant mouse colonization in competition with wild type, suggesting a role in the *in vivo* fitness of *V. cholerae* (Richard et al., 2010). However, regulation by TarA may not be equally critical in all *V. cholerae* strains since Bradley et al. (2011) did not observe difference in virulence comparing a *tarA* mutant strain with the wild type pandemic *V. cholerae* El Tor Biotype.

Also, it has been observed an increase in the mRNA *tcpF* in a *tarB* mutant, concluding that TarB negatively regulated the colonization factor TcpF and hence plays a negative role in virulence. Another study showed that TarB also decreases the expression of the transcription regulator VspR, which belongs to the *Vibrio* seventh pandemic island-1 (VSP-1; Bradley et al., 2011; Davies et al., 2012). VspR directly represses a gene that encodes a family of dinucleotide cyclases, which impacts virulence since the cyclic nucleotides play a role in biofilm formation, flagellum biosynthesis, DNA integrity and cell membrane stress. The signal molecule in *V. cholerae* seems to be predominantly cAMP-GMP, whose accumulation represses chemotaxis, and as an indirect effect enhances intestinal colonization by the bacterium (Davies et al., 2012).

Although most studies have been carried on *V. cholerae*, there are some studies that relate other *Vibrios* and regulation of virulence mediated by sRNA. In *V. parahaemolyticus* has been observed that the loss of Hfq provoked higher levels of *tdh* expression (which codes TDH hemolysin; Nakano et al., 2008). Other genes involved in the virulence of *V. parahaemolyticus*, such as VP1680, *vopC* and *vopt*, which are effectors secreted by the T3SS were also overexpressed in *hfq* mutant (Nakano et al., 2008; Nguyen and Jacq, 2014). These findings suggest that the expression of factors associated with the virulence of *V. parahaemolyticus* is negatively regulated by sRNAs, and Hfq may mediate the binding between these sRNAs and their target mRNA, thus affecting virulence.

Additionally in *V. parahaemolyticus* it has been shown that sRNA Spot 42 regulates the expression of the chaperone protein VP1682 in the T3SS1. Using electrophoretic mobility shift assay (EMSA), they proved that Spot 42 binds to the mRNA *vp1682* with the help of Hfq, and that the translation of this mRNA was inhibited *in vitro* in the presence of Hfq and Spot 42. In addition, in the deletion mutant of Spot 42 (Δ*spf*), an increase was seen in VP1682 and VP1680 expression compared to the parent strain. Cytotoxicity assays in infected Caco-2 cell culture showed that the mutant Δ*spf* has an increase in cytotoxicity compared to the wild type strain. These results show that Spot 42 regulates the expression of VP1682 post-transcriptionally, suggesting that it could contribute to the cytotoxicity of *V. parahaemolyticus in vivo* and constitute another example of the role of Hfq in the pathogenicity of these bacterial species (Tanabe et al., 2015).

The CRISPR/Cas system is another non-coding sRNA that plays an important role in virulence regulation. Sun et al. (2015) showed an association between the CRISPR sequence of 208 strains of *V. parahaemolyticus* and classical virulence factors of the species (*tdh and trh*). Of 153 *tdh*-positive strains, 149 isolates were identified as CRISPR positive, while 55 *tdh*-negative isolates were identified as CRISPR negative, suggesting a close connection between these components. These authors mentioned significant association of CRISPR with the *tdh* gene (Sun et al., 2013), suggesting an interesting role of sRNA in the evolution of pathogenesis. Li et al. (2014) analyzed the presence of CRISPR in 15 strains of *V. parahaemolyticus* of clinical and environmental origin. Using the CRISPR finder tool, the researchers found a total of six CRISPR elements. The results showed that environmental strains have fewer types of CRISPR than clinical strains, for which at least two CRISPR systems were found in each (Li et al., 2014).

## sRnas Implicated In Processes Related To Virulence In Human Pathogenic *Vibrio* spp.

### Quorum Sensing

Small non-coding RNAs can modulate the expression of genes associated with survival and virulence by pairing with untranslated regions of genes in response to cell density, through the QS pathway (Wen et al., 2016). QS is a process of cell communication by which a bacterial population expresses a set of genes in a coordinated way in response to the cell density in the population (Hammer and Bassler, 2007; Shao and Bassler, 2012, 2014; Van Kessel et al., 2015) through the production, release, detection and response to extracellular signal molecules called autoinducers (Tu and Bassler, 2007; Rutherford et al., 2011).

QS systems are widely distributed in *Vibrio* species (Zhang et al., 2012); the Qrr sRNA regulate multiple mRNA targets that code for QS regulatory components (Feng et al., 2015) through base-pairing with target mRNAs (Lenz et al., 2004; Svenningsen et al., 2008; Shao et al., 2013). These Qrr sRNAs appear in more than one copy per genome (Papenfort and Vanderpool, 2015). Qrr sRNAs activate the translation of AphA, a key regulator of low cell density (LCD), and suppress the translation of LuxR (*V. harveyi*)/ HapR (*V. cholerae)/* SmcR (*V. vulnificus*)/ LitR (*V. fischeri*)/ ValR (*V. alginolyticus*) and OpaR (*V. parahaemolyticus*), which is the main regulator of high cell density (HCD; Rutherford et al., 2011; Shao et al., 2013; Van Kessel et al., 2013b). Qrrs are highly conserved at the nucleotide level among *Vibrio* species but can vary in number and mechanism. For example, the five Qrr (Qrr1-5) of *V. harveyi* are additive because all of them are required to maintain wild-type-like repression of its master transcriptional regulator. Conversely, the four Qrr (Qrr1-4) of *V. cholerae* sRNAs are functionally redundant because any of its Qrr is sufficient to repress its master transcriptional regulator (Svenningsen et al., 2008). *V. vulnificus* has Qrr1-5 sRNAs that function additively, not redundantly, to repress SmcR (Wen et al., 2016). The only Qrr described in *V. fischeri* represses expression of the master regulator LitR (Kimbrough and Stabb, 2016) while in *V. parahaemolyticus* just three Qrr (Qrr2-4) sRNAs are known (Zhang et al., 2012). However, this does not imply that other Qrr could exist in these species. New Qrrs could be still discovered because of the large number of sRNA described each year.

Qrr sRNAs also give feedback to suppress genes that code one of the QS synthase-receptor pairs, LuxMN, and the gene that codes the LuxO transcription factor (Zhang et al., 2012; Feng et al., 2015). Phosphorylated LuxO activates the transcription of the Qrr sRNAs, which bind and destabilize the mRNA *hapR/luxR/smcR/opaR* as a function of Hfq (Tsou et al., 2011). However, the role of Qrr sRNA is not limited to QS, since they also regulate genes outside the QS circuit post-transcriptionally (Feng et al., 2015).

Although *V. harveyi* is not a human pathogen, is the species for which the QS process has been best characterized and has been considered as a general model for other *Vibrio* species, including human pathogens. In *V. harveyi* has been shown that Qrr3 controls its target mRNA through four different mechanisms, showing that a single sRNA molecule can play an important role in the regulation of its different targets by different mechanisms within the same bacterium, highlighting the versatility of these regulatory systems. In fact, Qrr3 sRNA suppresses *luxR* by catalytic degradation and *LuxO* by sequestering, in a process that does not lead to the degradation of Qrr. It also suppresses *luxM* by coupled degradation and activates *aphA* by revealing the ribosome binding site, while at the same time the sRNA is degraded (Feng et al., 2015). The activation of the translation of AphA (Rutherford et al., 2011; Van Kessel et al., 2013a) indirectly activates the transcription of virulence and biofilm genes, which promotes bacterial colonization and infection (Wang et al., 2014), so an important function of sRNAs in *Vibrios* is the link between QS and virulence (Carignan et al., 2016). Also, HapR/LuxR/SmcR/OpaR regulates the expression of genes involved in the synthesis and degradation of c-di-GMP reducing the levels of this second messenger that controls the switch between biofilm formation and motility and other fundamental bacterial behavior including fimbrial synthesis, T3SS and RNA modulation (Srivastava and Waters, 2012; Wang et al., 2014; Sengupta et al., 2016).

.

**FIGURE 1:**
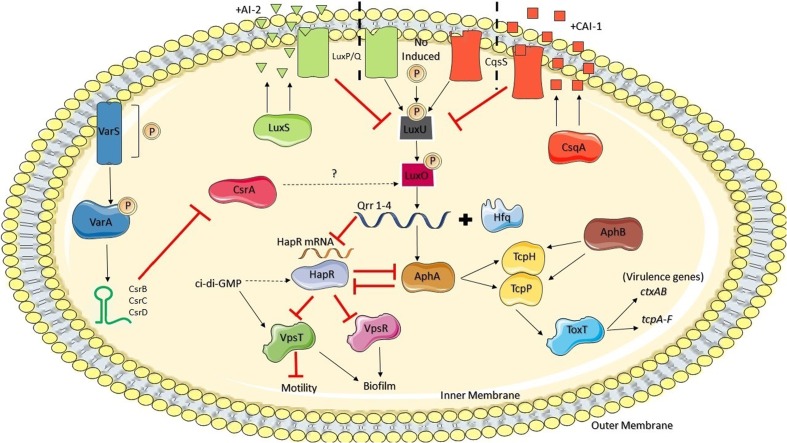
**Csr regulation circuit in *V. cholerae* and its relationship with HapR and Qrr sRNAs.** (Adapted from Vakulskas et al., 2015, with permission of the publisher)

In *V. cholerae*, sRNAs are critical components of the QS system and they constitute the first described example of sRNAs that regulate the expression of virulence genes in this pathogen (Tsou et al., 2011). There are sRNAs that participate directly in the expression of ToxT or are in turn controlled by ToxT, which is the main regulatory protein that controls the transcription of the genes *ctxAB* and *tcpA-F*, considered the main virulence factor genes of this pathogen (Carignan et al., 2016) (**Figure 1**). sRNAs that are members of the Qrr regulon indirectly control the production of the virulence factors TCP and CT through regulation of AphA and HapR (Bardill and Hammer, 2012; Shao and Bassler, 2014). HapR negatively regulates the expression of *aphA* and directly suppresses biofilm formation by binding to the promoter of the gene *vpsT*, which codes a regulator of the exopolysaccharide production cluster *vps*, and indirectly through *aphA*, since AphA is a positive regulator of *VpsT* (Teschler et al., 2015; Carignan et al., 2016) (**Figure 1**). HapR also suppresses the transcription of *vpsR*, which codes another transcription factor that also activates genes involved in biofilm formation and integrates signals from at least three different QS systems, which converge to the regulators of the LuxO response (Wang et al., 2014; Vakulskas et al., 2015) (**Figure 1**).

Notably, strains of *V. cholerae* modified to not produce Qrr sRNAs do not produce TCP or CT, and their capacity to colonize a lactating mouse model is severely lessened. HapR also positively regulates the transcription of *hapA*, a gene that codes the protease hemaglutinin (HA/protease), which is a virulence factor involved in the late stage of infection by *V. cholerae*, and the gene *prtV*, which codes a metaloprotease that is both a virulence factor against the human host and a protection factor against predators in the environment (Lutz et al., 2013). This system is reinforced by a mutual suppression in which HapR suppresses the transcription of *aphA* and AphA suppresses the transcription of *hapR*. Therefore, HCD induce the QS systems to reprogram gene expression, which stops the production of virulence factors and increases the production of transmission factors such as motility (Vakulskas et al., 2015).

Qrr sRNAs (Qrr1-4) act redundantly, and therefore the suppression of all four is required to avoid fully the repression of QS mediated by Hfq of genes downstream from HapR (Tsou et al., 2011). Moreover, in the absence of one or several Qrrs the others are overexpressed, compensating for the regulatory function. Svenningsen et al. (2008) observed that in *V. cholerae* HapR can activate the transcription of *qrr* genes, creating a negative feedback loop in the QS circuit. Therefore, HapR directly and indirectly suppresses its own production. Direct suppression of the promoter *hapR* by HapR only occurs at HCD, and it avoids overproduction of HapR at LCD. On the other hand, post-transcriptional suppression of *hapR* by the Qrr sRNA feedback loop requires the presence of LuxO-P and HapR, and therefore it only occurs in the transition from HCD to LCD. The latter feedback loop drastically accelerates the transition of *V. cholerae* cells from the social mode to the individual cell mode (Svenningsen et al., 2008).

Another case is the sRNA VqmR, which was described by Papenfort et al. (2015) in *V. cholerae* through a differential RNA-seq. VqmR regulates biofilm formation by suppression of *vpsT*, which is controlled by QS through HapR, revealing that VqmR represents a previously unknown link between biofilm formation and QS. VqmR suppresses the expression of multiple mRNAs, including those that code for the toxin Rtx, and *vpsT*, which is required for biofilm formation, as demonstrated by the researchers through mutagenesis and microarray analysis (Papenfort et al., 2015). Shao and Bassler (2014) showed that LuxO functions through the sRNAs involved in QS to suppress T6SS, a new virulence factor in *V*. *cholerae* (Shao and Bassler, 2014; Cheng et al., 2015), using two mechanisms: the Qrr sRNAs suppress the production of T6SS-activator HapR, thus decreasing the expression; independently of HapR, the Qrr sRNAs suppress T6SS by direct base-pairing with the mRNA that codes the long T6SS cluster. Regulation of T6SS Qrr sRNA is also conserved in pandemic and non-pandemic strains of *V. cholerae* (Shao and Bassler, 2014).

**FIGURE 2:**
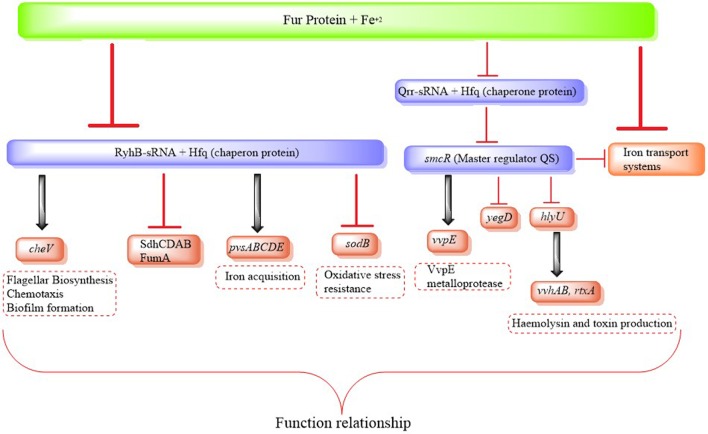
**Regulatory chain of Fur/RyhB mediating the regulation of virulence determinants in environments with low iron availability**.

There is also a relation between virulence and QS in other human pathogen *Vibrios*. The expression of virulence factors in *V. parahaemolyticus* is also modulated by QS, allowing differential genetic regulation under LCD and HCD conditions. OpaR controls opacity, biofilm formation and motility, represses T3SS1 regulons and oppositely, regulates the two T6SS in this bacterium (Gode-Potratz and McCarter, 2011; Burke et al., 2015). This QS-dependent regulation of T6SS is crucial as this system plays an important role in pandemic and non-pandemic strains of *V. parahaemolyticus* (Ceccarelli et al., 2013). Similarly, Qrr sRNAs affect the expression of pathogenicity genes in *V. vulnificus* by modulating the expression of the *smcR* (**Figure 2**). The quorum-sensing pathway is similar to *V. cholerae*, and SmcR is responsible for regulating the expression of diverse virulence factors (Wen et al., 2016). *smcR* mutants show attenuated cytotoxicity compared to the wild type (Lee et al., 2007). Wen et al. (2016) showed that Qrr sRNAs in *V. vulnificus* are regulated via LuxO and modulate the expression of virulence factors via SmcR. Interestingly, the authors also showed that Qrr sRNAs are responsive to iron concentration, and that iron and QS converge on Qrrs to control the expression of virulence factors (Wen et al., 2016) (**Figure 2**).

### The CsrA/RsmA Pathway and Its Implication on QS

The CsrA/RsmA pathway has been identified in several γ-proteobacterial species, in which it regulates a wide range of cellular physiology: regulation of carbon metabolism, motility, biofilm formation, production of secondary metabolites and QS, thus affecting virulence (Babitzke and Romeo, 2007).

Maturation and dispersal of biofilms in *V. cholerae*, as well as CT and TCP production, are controlled by multiple systems independent of QS whose effects converge on a single regulator circuit (Rutherford and Bassler, 2012; Mey et al., 2015). CsrA influences the expression of virulence factors and QS through this circuit, connecting the response to autoinducers CAI-1 and AI-2 (Mey et al., 2015) (**Figure 1**). VarS/VarA (Virulence Associated Regulator) regulate QS indirectly through CsrA and three CsrA-inhibiting sRNAs: the aforementioned CsrB and also CsrC and CsrD (Lenz et al., 2005) (**Figure 1**). The response regulator VarA has been identified as a virulence regulator in this bacterium (Tsou et al., 2011). VarS/VarA has also been found to be involved in the control of HapR expression through LuxO and in the control of the CqsS and LuxPQ systems (**Figure 1**). Lenz et al. (2004) observed that Hfq is required for the regulation of QS due to the dependent chain of LuxO and that *hfq* mutation affects the stability of the *hapR* messenger. Therefore, the VarS/VarA-CsrA/CsrBCD system converges with the QS system in *V. cholerae* to regulate the expression of the Qrr sRNAs and thus the entire QS regulon (Lenz et al., 2004). All these pathways converge to HapR, which thus represents a central node in this regulation network (**Figure 1**).

### Iron Metabolism

A fundamental element of homeostasis in microorganisms is iron, which is a cofactor of a large number of enzymes involved in essential biological processes (such as photosynthesis, respiration, DNA biosynthesis, etc.; Mey et al., 2005b; Repoila and Darfeuille, 2009; Porcheron and Dozois, 2015). However, excess free intracellular iron will give rise to reactions that generate toxic oxygen-reactive species (Nguyen and Jacq, 2014). Therefore internal iron levels must be rigorously controlled in accordance with physiological requirements, thus avoiding toxic effects.

Bacteria respond to fluctuations in iron concentration by coordinating the expression of genes that code for proteins involved in iron transport and storage, and genes that code for iron-dependent enzymes (Repoila and Darfeuille, 2009; Nguyen and Jacq, 2014). In Gram-negative bacteria, the main repressor of iron uptake systems is the ferric uptake regulation (Fur) protein. Fur complexed with Fe^2+^ binds to DNA (Bagg and Neilands, 1987), turning off the transcription of the iron uptake genes and limiting the entry of excess iron into the cell. When iron is limited in the cell, Fur is inactivated by the release of the iron cofactor, and the iron uptake genes are transcribed. Fur also regulates other genes involved in general metabolism and pathogenicity, thus the availability of iron in the host constitutes a key signal which coordinately regulates the expression of those genes (Tanabe et al., 2013).

Uncommonly, *E. coli fur* mutants have significantly reduced levels of total cellular iron despite the constitutive expression of iron uptake pathways (Abdul-Tehrani et al., 1999). This apparent paradox was explained by the presence of a sRNA, RyhB, which regulates the repression of numerous iron-containing proteins (Massé and Gottesman, 2002). In the absence of Fur, the RyhB sRNA have a key role in the control of iron homeostasis, repressing the synthesis of succinate dehydrogenase, aconitase, fumarase and iron superoxide dismutase. Total cellular iron is decreased as a result, despite a significant increase in cytosolic iron. Fur represses the synthesis of the RyhB, and RyhB in turn negatively regulates the synthesis of proteins that bind iron in the cell (Massé and Gottesman, 2002).

The mechanism of regulation by RyhB is post-transcriptional, requires Hfq and involves base pairing between RyhB and its mRNA target and subsequent RNase E-mediated degradation of the RyhB-mRNA duplex (Massé et al., 2003). Porcheron and Dozois (2015) distinguished three direct and indirect regulation mechanisms of virulence determinants by Fur and RyhB in response to environmental iron concentration. Fur and RyhB can act directly, regulating the expression of a gene, as is the case with genes involved in iron acquisition that are directly suppressed by Fur. The second mechanism involves other transcriptional regulators that determine invasion, acid resistance and toxin production; hence Fur/RyhB would indirectly regulate virulence. The last mechanism is related to the regulation of the locus of enterocyte effacement genes in *E. coli*, which occurs through the transcriptional regulator Ler; however, neither Fur nor RyhB directly regulates the expression of Ler (Porcheron and Dozois, 2015).

RyhB has a conserved region between *E. coli* and *V. cholerae*. The sRNA is longer in the latter, reaching lengths of ∼60 nt on each side of the conserved region (Porcheron et al., 2014). Despite the conserved region, the two sRNAs differ in the degree and function of their regulons, since various mRNAs encoding iron-containing proteins in *E. coli* are similarly regulated by RyhB in *V. cholerae*, but other transcripts regulated by RyhB in *E. coli* showed no RyhB-dependent regulation in *V. cholerae* and vice versa (Updegrove et al., 2015). Thus in both species RyhB has a core function for iron homeostasis, but in *V. cholerae* the sRNA has acquired new physiological roles (Updegrove et al., 2015).

RyhB is regulated by Fur in *V. cholerae*, and interacts with the protein Hfq, required for the suppression of genes that code for the SodB superoxide dismutase and several enzymes in the tricarboxylic acid cycle, including succinate dehydrogenase SdhCDAB and fumarate reductase FumA (**Figure 2**). RyhB also modulates the expression of several genes that control motility, chemotaxis and biofilm formation in *V. cholerae*, since several genes involved in flagella biosynthesis and chemotaxis are negatively regulated in a *ryhB* mutant of *V. cholerae*, and as mentioned above these are important processes in the virulence of the microorganism (Davis et al., 2005; Mey et al., 2005a) (**Figure 2**). Both Hfq and RyhB can be co-purified from *V. cholerae* and high levels of RyhB are only detectable in the presence of Hfq, suggesting that as with *E. coli*, Hfq stabilizes this sRNA (Davis et al., 2005). Using microarray analysis, Mey et al. (2005b) showed that numerous genes that are involved in iron acquisition, including *fhuAC*, *feoAB* and *irgA*, were increased when RyhB was expressed. In addition, a *fur* mutant of *V. cholerae* was unable to use pyruvate, succinate or fumarate as carbon sources (Litwin and Calderwood, 1994; Mey et al., 2005a). This suggests that *V. cholerae* may use a system analogous to the *E. coli* RyhB mechanism for regulating genes encoding iron-containing proteins and those involved in iron metabolism.

Homologs of *V. cholerae* RyhB have been identified in other *Vibrio* species including *V. vulnificus* and *V. parahaemolyticus* (Mey et al., 2005a; Porcheron et al., 2014). In *V. vulnificus* iron is necessary for growth and increased host mortality *in vivo* (Wen et al., 2016). Alice et al. (2008) carried out competitive experiments in an “iron-overloaded mouse” model with the wild type strain of *V. vulnificus*, which showed that the mutant *ryhB* is less virulent. While the *fur* mutant (lethal dose) LD_50_ is the same as the wild type strain, the LD_50_ of the RyhB mutant is more than 30 times greater than that of the wild type strain (Alice et al., 2008; Porcheron and Dozois, 2015). The researchers attributed the attenuated virulent phenotype of the *V. vulnificus ryhB* mutant to a defect in the growth observed in this mutant under limited iron conditions. However, in a double mutant Δ*ryhB fur*::pDM4 Δ*lacZ* growth was recovered under limited iron conditions, although the virulence remained attenuated, possibly implying that other genes related to the virulence of *V. vulnificus* may be directly or indirectly under the control of RyhB (Alice et al., 2008).

*Vibrio parahaemolyticus* can use several siderophores, including an analog of vibrioferrin, exogenous aerobactin, ferrichrome and enterobactin to acquire iron under low iron conditions (Tanabe et al., 2013). It has been observed that the production of vibrioferrin decreased in a *ryhB* deletion mutant of *V. parahaemolyticus* compared to the parent wild type strain. This suggests that RyhB positively regulates the production of vibrioferrin, and the evidence indicates that this probably occurs through Hfq-dependent stabilization of the operon *pvsOpm* which is involved in its biosynthesis. This was the first report to state that RyhB positively regulated a polycistronic mRNA (Tanabe et al., 2013).

## Bioinformatics Analysis Applied To The Study Of sRnas

The continuous discovery of new sRNAs and their potential action mechanisms has increased the interest in identifying associations between sRNA and bacterial pathogenesis (Papenfort and Vogel, 2010). Computational predictions conducted with the enormous number of microbial genomes currently available and experimental tracing using new technologies such as microarrays and RNA-seq have discovered an unexpected number of RNA loci (Papenfort and Vogel, 2010; Pain et al., 2015). Silveira et al. (2010) used a combination of *in silico* searches validated by microarray analysis and RT-PCR to find and characterize a diverse range of sRNAs in the genomes of *V. alginolyticus*, *V. campbellii*, *V. mimicus* and *V. communis*. This approach culminated in the identification of 31–38 genes encoding putative sRNAs per species (Silveira et al., 2010).

TargetRNA is a bioinformatics tool designed to predict mRNA targeted by the action of sRNA in bacteria through base complementarity. It has been validated experimentally in *E. coli* using both northern blot and microarray experiments (Tjaden, 2008). The predictions of this program have also been corroborated in *V. cholerae*, in which several sRNAs have been identified using gene tracing and computational methods (Song and Wai, 2009). A more recent actualization of TargetRNA2 includes new characteristics such as conservation of sRNAs in other bacteria, secondary structure of the sRNAs, secondary structure of each possible target mRNA and the hybrid energy between the sRNA and the target mRNA (Kery et al., 2014). Sabharwal et al. (2015) performed an *in silico* analysis with TargetRNA to identify new VrrA-targeted mRNAs, finding *vrp* as a possible sRNA target. This prediction was complemented with another tool, RNAhybrid, to authenticate the pairing region between VrrA and *vrp* (Sabharwal et al., 2015). Another tool is the software package sRNAPredict. Davis and Waldor (2007) used this program to identify the sRNA MicX. This sRNA negatively regulates the expression of *vc0972*, which encodes an uncharacterized OMP, and vc0620, which encodes the periplasmic component of a peptide ABC transporter in *V. cholerae*. This prediction was made based on the proximity of a potential rho-independent terminator of intergenic sequences conserved between *V. cholerae*, *V. parahaemolyticus* and *V. vulnificus* (Davis and Waldor, 2007). More recent tools to detect sRNA such as CopraRNA and IntaRNA were described by Wright et al. (2014). CopraRNA is able to use the whole genome available on platforms (NCBI, Rfam) to obtain three or more sequences of homologous sRNA for a set of given organisms. In contrast, IntaRNA can also be applied to non-whole genome screens using smaller sets of RNA molecules as input, in the absence of homologous sRNA. It is focused on the prediction of interactions between two RNA molecules and domain interaction. Both complementary results should generally be evaluated, because this allows characterizing the binding site region of the target (Wright et al., 2014).

The main challenge in the study of new sRNAs is not just their identification, but also elucidating their targets (Kery et al., 2014). sRNAs-*trans* normally have limited complementarity with their target mRNAs (Kery et al., 2014) and sRNA-mRNA hybrids occur in relatively short regions that are not easily distinguishable from many other hybrids formed by random pairs of transcripts (Pain et al., 2015). Because of these limitations, predicting the regular function of sRNAs through bioinformatics identification of their target mRNAs has proven difficult (Song and Wai, 2009). Besides, the use of prediction programs presents a series of problems, as described by Pain et al. (2015), who evaluated sRNA target prediction programs using reliable sRNA/target pairs of *E. coli*. They noted that despite continuous improvements in computer programs, the number of true biological targets with low scores (false negatives) and the non-targets with high scores (false positives) remains counterproductive. This may be partly because many true targets have yet to be validated experimentally. sRNA-mRNA interaction *in vivo* can be positively affected by the presence of sRNA chaperones such as Hfq, which can significantly influence computer predictions. Another reason is that most programs predict possible targets based on free energy values of binding. However, high binding energy does not necessarily imply efficient processing of the target mRNA by the Hfq-sRNA complex (Pain et al., 2015).

## Conclusion

Several studies regarding sRNAs in the full genomes of bacteria carried out in recent years have revealed that they are highly variable, widely distributed and of diverse functionality. It has also been shown that they play a crucial role in many biological processes such as development, metabolism, adaptation to stress, QS and virulence. The discovery of new sRNAs in the family *Vibrionaceae*, particularly in pathogenic species, and the knowledge of the targets they regulate is continuously increasing. Thus establishing a connection between the regulation mediated by sRNAs and virulence would shed new light on the understanding of the mechanisms of infection in these species. Despite the difficult task of identifying the targets regulated by sRNAs, the growing number of sRNAs identified together with their target genes in a wide range of bacteria has allowed comparisons between species (Updegrove et al., 2015). Although most studies in the *Vibrionaceae* have focused on *V. cholerae*, several laboratories are working on the implications of sRNAs in the virulence mechanisms of other human pathogenic species of this family. In this review we discussed some well characterized sRNAs, but there are many more sRNA described in databases whose function is unknown, and consequently much information remains to be elucidated. Currently most sRNAs in *V. parahaemolyticus* and *V. vulnificus* are described only by homology, thus experimental validation is highly required. Even though the bioinformatics tools are increasingly advanced, they are just an initial approximation in the study of sRNA and its targets, but the interaction between the mRNA-sRNA complex and the sRNA mechanisms of action can only be verified with experimental techniques, including EMSA, mutant development and RNA expression assays (such as microarrays and RNA-seq). Additionally, the fact that there are some contradictory effects in the function of one sRNA depending on the strain used calls for caution when these results are extrapolated to other species. Thus although there has been much progress in the study of sRNA in *Vibrionaceae*, more experimentation is needed to determine with certainty which sRNAs govern the virulence mechanism of human pathogenic *Vibrio*.

## Author Contributions

DP-R and KG conceived the idea and wrote the manuscript. NP made the figures. RE wrote the bioinformatics item. PN and RB wrote about CRISPR and *Vibrio vulnificus* items, respectively. All authors read and approved the final version.

## Conflict of Interest Statement

The authors declare that the research was conducted in the absence of any commercial or financial relationships that could be construed as a potential conflict of interest.
